# The contribution of CD200 to the diagnostic accuracy of Matutes score in the diagnosis of chronic lymphocytic leukemia in limited resources laboratories

**DOI:** 10.1371/journal.pone.0247491

**Published:** 2021-02-19

**Authors:** Sana Dlawar Jalal

**Affiliations:** Department of Pathology, College of Medicine, University of Sulaimani, Sulaimani, Iraq; European Institute of Oncology, ITALY

## Abstract

Flow cytometry immunophenotyping has an essential role in distinguishing chronic lymphocytic leukemia from other B-chronic lymphoproliferative disorders. Recently, CD200 is considered as a relatively consistent marker in chronic lymphocytic leukemia. We retrospectively assessed CD200 expression in 252 patients with B chronic lymphoproliferative disorders with four-color flow cytometry. CD200 expression estimation included the proportion of positive cells (≥30%) and the mean fluorescence intensity ratio. Additionally, we have incorporated CD200 into Matutes score, also replaced FMC7 and CD79b in an attempt to improve the score discriminative power. Of 252 patients enrolled, 199(79%) patients were classified as chronic lymphocytic leukemia and 53 (21%) as other B-chronic lymphoproliferative disorders. All chronic lymphocytic leukemia cases and 20 of 53 (37.7%) of other B-chronic lymphoproliferative disorders demonstrated high CD200 expression (≥30%). Further, CD200 (≥30%) revealed a higher accuracy in comparison to other markers in Matutes score (range: 51%–92.5%). Also, CD200 addition to the Matutes score has correctly recognized all 199 chronic lymphocytic leukemia cases including 10 atypical chronic lymphocytic leukemia cases. As for non-CLL cases, 20 of 53 attained a higher score, yet keeping the original diagnosis. Moreover, CD200 enhanced the diagnostic accuracy of Matutes score to 100%, and when included in a simplified 4-markers score, showed an accuracy of 99.8% compared to 99.4% of Matutes score. In conclusion, CD200 is an accurate diagnostic marker for chronic lymphocytic leukemia, and can refine the modified Matutes score accuracy when added with other markers.

## Introduction

Chronic lymphocytic leukemia (CLL) is a clonal expansion of monomorphic, mature, immunologically incompetent CD5+ B-cells in peripheral blood, bone marrow, and secondary lymphoid organs. It’s considered the most common leukemia in adults with a male predominance and an average age at presentation of 72 years. The incidence is10 to 20 times less in Asia than in Western countries suggesting the influence of some genetic and environmental factors on disease susceptibility [1,2]. Diagnostic accuracy is of utter importance as the therapeutic options differ significantly for various B-chronic lymphoproliferative disorders (B-CLPD). Chronic lymphocytic leukemia diagnosis requires the presence of ≥ 5 x10^9^/l monoclonal B-cells exhibiting characteristic immunophenotype initially described by Estella Matutes” Matutes scores MS” [3] [surface membrane immunoglobulin (SmIg) weak, CD5+, CD19+, CD23+, CD22 weak/-, and FMC7 –], that was modified later by replacing CD22 by CD79b [4]. In Matutes scoring system, a value of 0 or 1 is assigned according to the expression of the abovementioned five markers. The majority of CLL cases have a score of 4 or 5 whereas non-CLL cases score below 4. Some atypical CLL cases may score 3 [5,6] where the addition of more markers such as ROR1 [7], CD81 [8], CD43 [9], and most importantly CD200 would be very helpful [10]. CD200, a glycoprotein on the surface membrane of normal B-cells, B-cell precursors, some T-cells, dendritic cells, and neurons [11], was first described in 2009 for being uniformly expressed in CLL, but absent in mantle cell lymphoma (MCL) [12]. Since then, it’s potential role had expanded and considered as valuable as CD23 in the diagnosis of B-CLPD [13]. This study, therefore, has investigated the diagnostic benefit of adding CD200 to MS in a large Iraqi single-center cohort of 252 patients with chronic B-cell lymphoproliferative disorders. Further, we assessed the performance of the 4-marker score; CD5/CD23/CD200/sIgM, in optimizing the accuracy of CLL diagnosis in resource constrained laboratories.

## Materials and methods

### Patients

The current retrospective analysis started on 12/1/2020 including a total of 195 peripheral blood and 57 bone marrow aspirates and/or bone marrow biopsies of patients with suspected B-CLPD processed at Sulaimani Public Health Laboratory, Sulaimani-Kurdistan/Iraq over the period from January 2012 to December 2019. The patients aged between 35-87years old with a median of 63 years, 184(73%) were males and 68(27%) were females, with a male-to-female ratio of 2.7:1. All enrolled cases were referred from hemato-oncology hospitals from different provinces in Iraq and the study sample can be considered representative of a larger population. The diagnosis was determined by results from diagnostic procedures including; cytomorphology, flow cytometry (FC), and immunohistochemistry (IHC) according to the World Health Organization (WHO) guidelines and International Working Group on CLL (IWCLL) [14,15]. The inclusion criterion was as follows; Patients with full database; demographic and laboratory data including a set of flow cytometry markers necessary for B-CLPD diagnosis.

### Immunophenotypic analysis

All specimens, freshly collected in K3 EDTA tubes, were stored at 4C^0^, stained and analyzed within 24–36 hours from collection using a direct immunofluorescence method, as detailed below: A total of 1 × 10^6^ cells from whole blood samples were incubated for 15 minutes in the dark at 37C^0^ with monoclonal antibodies (MAbs) targeting the antigens: CD5, CD23, CD19, CD20, CD10, FMC7, CD79b, CD200, sIgM, Kappa, Lambda, CD38, CD103, CD11c, CD25, and CD123. Isotype antibodies were used as a negative control in separate tubes. All MAbs were purchased from BD Biosciences (Fluorochromes and clones are outlined in S1 Table). Cells were lysed within 5 minutes by (Becton Dickinson (BD) FACS Lysing solution and washed in Phosphate Buffer Solution (PBS). Thereafter, cells were re-suspended in 500 μL of PBS and immunophenotyping analysis was performed using two lasers, four-color, six-parameter BD FACS Calibur flow cytometer (BD Biosciences, San Jose, CA, USA). Data acquisition and analyses were performed using CellQuest Pro software (BD Biosciences). Calibration was performed using BD CaliBRITE beads with daily quality control to preserve the reproducibility of the fluorescence intensities. At least 10,000 gated events were acquired and the lymphocytes population was selected by gating on CD45^high^/sideways scatter (SSC) ^low,^ and subsequent analysis was carried out on CD19+ cells. The modified Matutes score was calculated as described earlier [4], and positivity was established as≥ 30% positive cells population [6,16–18]. CD5 and CD23 were counted score 1 when the positive cells population was ≥30%, while FMC7 and CD79b were considered score 1 when the positive cells population was <30%. Additionally, sIgM was considered score 1 when the expression was weak. Typical CLL cases were defined by a score ≥ 4 and atypical cases were identified by a score < 4. Likewise, CD200 was designated score 1 when the positive cells population was ≥30%. CD200 expression intensity was estimated as mean fluorescence intensity ratio (MFIR) (MFI sample/MFI isotype) with a positivity threshold set up at 18 according to a prior study [6] (S1 Fig). Furthermore, the expression pattern (weak, moderate, or high) was evaluated according to the log scale of the fluorescence axis on the B-cell population. Accordingly, weak expression if the positive peak lied within the first logarithmic percentile, moderate in the second percentile, and strong when it was above the second percentile [19].

### Ethical consideration

The study was approved by the ethical committee at Sulaimani College of Medicine, University of Sulaimani, Iraq. All methods were performed in accordance with the Helsinki Declaration and verbal informed consents were obtained from all enrollees and documented within laboratory database.

### Statistical analysis

SPSS version 25.0 (Armonk, NY: IBM Corp, USA) was used to carry out the analysis. For evaluating the performance of diagnostic tests and the accuracy of a statistical model, the sensitivity and specificity were measured using the ROC test, and optimal cut-values have been chosen as to achieve a maximum sensitivity and specificity. The sensitivity and specificity of CD200, as a percentage of positive cells, were calculated at an optimum cut value of 88%, while the cut value for CD200 MFIR was 36.8%. A score of ≥4 was selected to evaluate CLL diagnosis in the classical and currently modified Matutes Scores, and MS was considered as a comparator with the modified MS. McNemar’s test was used to estimate statistically significant differences between MS systems. Additionally, continuous variables were compared using Mann–Whitney’s U test. *P* values of 0<0.05 were considered statistically significant.

## Results

The immunophenotypic classification of 252 patients with a provisional diagnosis of B-CLPD is as follows: 199 cases classified as CLL (79%), and 53 cases were non-CLL (21%) [20 mantle cell lymphoma (MCL), 17 marginal zone lymphoma (MZL),8 hairy cell leukemia (HCL),3 follicular lymphomas (FL)and 5 other B-CLPD [2 lymphoplasmacytic lymphomas (LPL),1Waldenstrőm macroglobulinemia (WM), and 2 prolymphocytic leukemia (PLL)], (Table 1).

**Table 1:** Diagnosis of 252 cases with suspected B-chronic lymphoproliferative disorders.

Patients(n = 252)	No.	%
**CLL**	199	79
**Mantle cell lymphoma (MCL)**	20	7.9
**Marginal zone lymphoma (MZL)**	17	6.7
**Hairy cell leukemia (HCL)**	8	3.2
**Follicular lymphoma (FL)**	3	1.2
**Other B-CLPD**	5	2

Of the total CLL cases, 189 / 199 CLL cases had the typical CLL phenotype (MS ≥4), while 10 cases scored 3 and diagnosed as atypical CLL (with negative cyclin D1 by IHC on LN biopsy or BM biopsy). Details of atypical CLL cases are provided in (S2 Table). All the B-CLPD cases with MS between 0–3 required correlation with clinical data and another pathology testing including, histological and where available IHC. Cases with typical morphology and immunophenotypic markers expression (e.g. CD103, CD25, CD11c, and CD123) were regarded as convenient for HCL diagnosis without further histopathological confirmation. Regarding non-CLL B-CLPD, 46/53(86.8%) had MS of 0–2 (Table 2).

**Table 2:** CD200 expression in B-cell chronic lymphoproliferative disorders.

CD200 expression							
	Median percentage (range)	n, CD200 ≥ 30%	n, CD200 < 30%	Median MFIR (range)	n, CD200 MFIR ≥ 18	n, CD200 MFIR < 18	Matutes score (n patients)
**CLL (n = 199)**	97.9(50.6–100.0)	199 (100%)	0	59.0 (39.3–69.9)	199 (100%)	0	3(10), 4(95), 5(94)
**HCL (n = 8)**	46.4 (0.9–91.8)	5 (62.5%)	3 (37.5%)	46.5 (1.5–77.0)	5(62.5%)	3 (37.5%)	0(1), 1(3), 2(3)3(1)
**MZL (n = 17)**	55.9 (0.4–88.0)	12 (70.6%)	5 (29.4%)	24.0 (1.3–58.0)	12 (70.6%)	5 (29.4%)	0(7), 1(4), 2(4), 3(2)
**MCL (n = 20)**	2.3 (0.1–41.0)	0	20 (100%)	3.3 (0.7–43.0)	0	20 (100.0%)	1(10), 2(7), 3(3)
**FL (n = 3)**	1.7 (0.9–70.6)	1 (33.3%)	2 (66.7%)	3.0 (1.6–43.0)	1 (33.3)	2 (66.7%)	2(2), 3(1)
**Others (n = 5)**	5.9 (1.9–57.6)	2 (40.0%)	3 (60.0%)	1.5 (1.0–45.0)	2 (40.0%)	3 (60.0%)	0(1), 1(1), 2(3)

**Fig 1:**
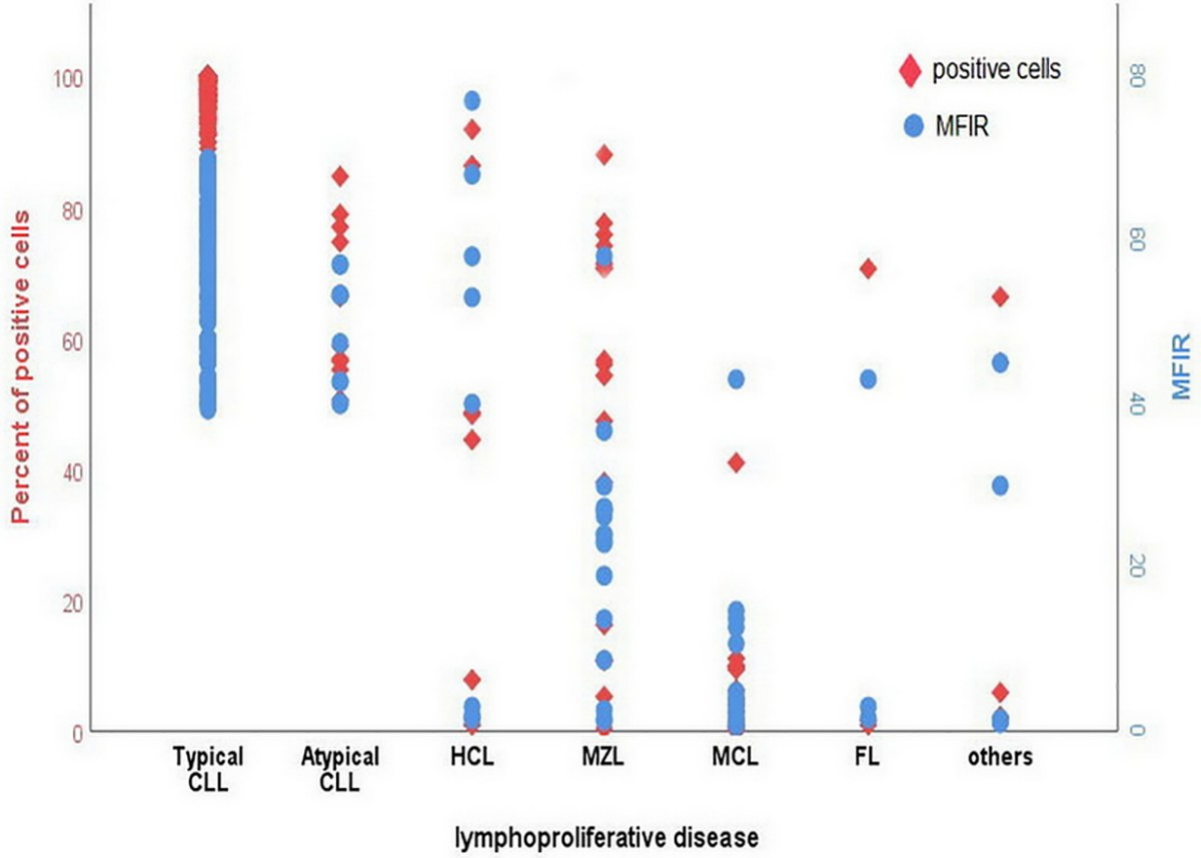
CD200 expression levels (percent of positive cells and MFIR) on 252 B- chronic lymphoproliferative disorders enrolled in this study.

**Fig 2:**
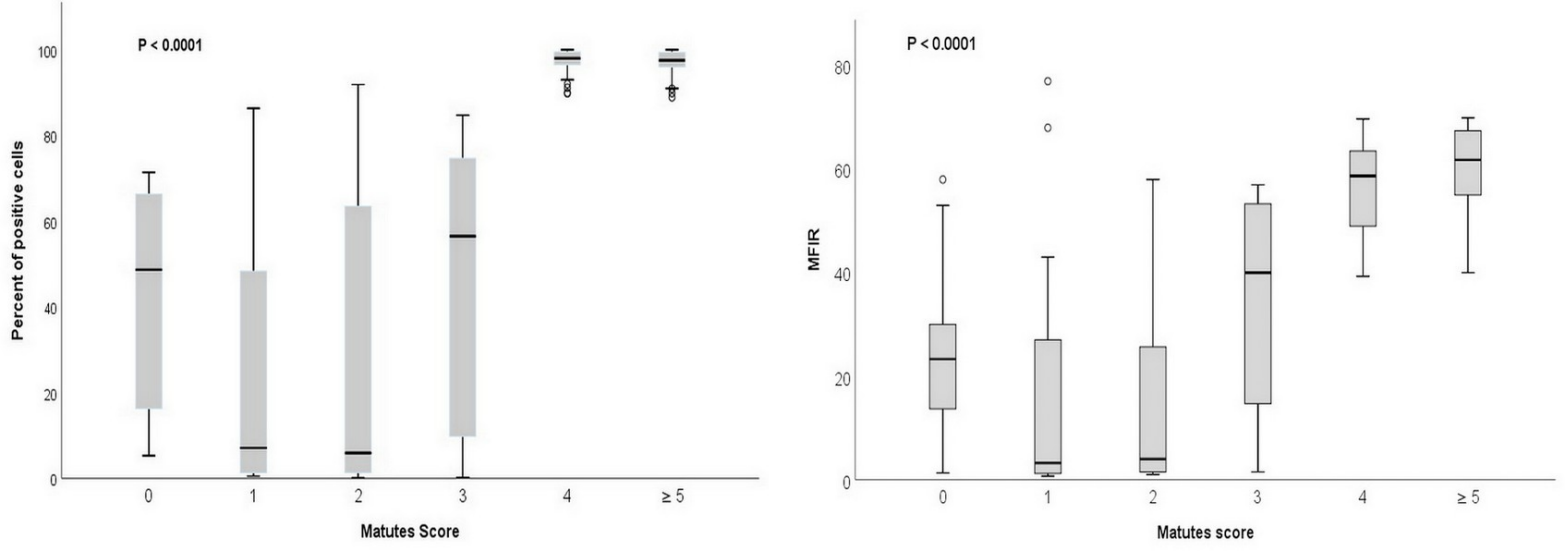
CD200 expression level on B-chronic lymphoproliferative disorders classified by Matutes score. (A) Shows median, minimum and maximum levels of CD200 percentage of expression. (B) Shows median, minimum and maximum levels MFIR of CD200.

In CLL cases, CD200 positive B- cells (≥ 30%) were detected in all cases [median 97.9% (50.6–100)] as opposed to a highly heterogeneous CD200 expression pattern in the non-CLL group, reported in 37.7% of cases, [median 9.8 (0.10–91.8%)],p value<0.0001 (Table 2 and Fig 1). Among non-CLL cases, the highest CD200 values were seen in MZL (median 55.9%), followed by HCL (median 46.4%), while all MCL cases were negative for CD200. Similarly, all 199 CLL cases displayed a potentially higher MFIR of CD200 expression [median MFIR of 59 (39.3–69.9) versus 8.7 (0.7–77) in non-CLL group (P < .0001)] (Table 2 and Fig 1). Further, CD200 expression (MFIR ≥ 18.0); was seen in all cases of CLL and in 20/53 (37.7%) cases from non-CLL group; including 5 of 8 HCL cases and 12 of 17 MZL but non from MCL group. Additionally, atypical CLL group displayed less intense CD200 expression opposed to classical CLL; as proportion of positive cells ≥ 30% [median 62.6(50.6–84.7%)] vs. [median 98(88.9–100)] and MFIR [median 50.3 (40–57)] vs median 59.8(39.3–69.9)], respectively, p value <0.001. Of note, a higher CD200 expression (CD200 ≥30% and MFIR) correlated significantly to higher MS scores p<0.0001 (Fig 2A and 2B).

### Diagnostic utility of CD200 in CLL diagnosis

As a diagnostic marker, CD200 (≥30%) revealed a sensitivity of 94.97%, a specificity of 98.11%, with considerably higher accuracy than other MS markers (CD5, CD23, FMC7, CD79b and sIgM) (99.1%) in CLL diagnosis, p value<0.001, (Table 3). This is at variance to CD200 expression evaluated as MFIR (≥18.0), where a lower accuracy (93.6% vs. 99.11%, respectively), a higher sensitivity (100% vs. 94.97%, respectively), and lower specificity (83.2% vs. 98.1%, respectively) were detected with a significant difference in CLL discriminative accuracy, p < 0.04. Expectedly, CD5, and CD23 were informative in this analysis, in contrast to FMC7 and CD79b markers that didn’t yield a high diagnostic value. Likewise, sIgM showed a low specificity in CLL diagnosis (Table 3).

**Table 3:** Sensitivity, specificity, and accuracy of individual markers in the differential diagnosis of CLL.

Marker	Matutes Score				*Sensitivity % (95% CI)*	*Specificity %*, *(95% CI)*	*CLL vs*. *non-CLL % (95% CI)*	P value
**CD200**^**a**^	Negative	0	positive	1	94.97 (91.0 - 97.6)	98.11 (89.9 - 100.0)	99.1 (96.9–99.9)	< .001
**CD200**^**b**^	Negative	0	positive	1	100.0 (98.2 – 100.0)	83.02 (70.2 - 91.9)	93.6 (89.8–96.3)	< .001
**CD5**^**a**^	Negative	0	positive	1	100.0 (98.2–100.0)	62.26 (47.9–75.2)	81.1 (75.7–85.8)	< .001
**CD23**^**a**^	Negative	0	positive	1	98.49 (95.7–99.7)	79.25 (65.9–89.2)	88.9 (84.3–92.5)	< .001
**FMC7**^**c**^	Negative	1	positive	0	93.97 (89.7–96.8)	20.75 (10.8–34.1)	57.4 (51.0–63.5)	< .05
**CD79b**^**c**^	Negative	1	positive	0	52.26 (45.1–59.4)	58.49 (44.1–71.9)	55.4 (49.0–61.6)	NS
**sIgM**^**d**^	Weak	1	Moderate/Strong	0	87.44 (82.0–91.7)	52.83 (38.6–66.7)	70.1 (64.1–75.7)	< .001

aCD200 was regarded score 1 when the positive B -cell population was ≥30%.

bCD200 was regarded score 1 when the positive B-cell population was MFIR ≥18.

c FMC7 and CD79b were regarded score 1 when the positive B-cell population was <30%.

d sIgM was regarded score 1 when the expression was weak.

NS = Non Significant P > 0.05.

### Modification of Matutes score

The addition of CD200 (≥30%) to MS has modified the score in 30 of 252 cases included in the study. All 10 cases of atypical CLL (MS 3) were re-categorized into MS 4(i.e., classical CLL). Likewise,20 cases in the non-CLL group had a modified higher score; including 1 HCL case (from MS 3 to MS 4) and 9 from non-CLL cases into MS 3 (4 from MZL, 2 HCL,2PLL and 1 from FL), (Table 4). Also, the simple four-marker MS including CD200 together with CD5, CD23 and sIgM, has categorized 94% of CLL cases as classical CLL (≥4), whereas 12 cases have a MS of 3. Additionally, 4 cases from other B-CLPD (2MZL, and 2PLL cases) scored 3 in the 4-marker scoring system.

**Table 4:** Patients classification according to Matutes score and the alternative proposed score.

Classical MS			MS with CD200 added		MS with CD200replaced FMC7 &CD79b	
MS	CLL	Non-CLL	CLL	Non-CLL	CLL	Non-CLL
**≥5**	94	0	189	0	0	0
**4**	95	0	10	1	187	0
**3**	10	7	0	16	12	4
**2**	0	19	0	19	0	16
1	0	18	0	14	0	30
0	0	9	0	3	0	3

Interestingly, the addition of CD200 to CD5, CD23, CD79b, FMC7, and sIgM has improved the accuracy and sensitivity of the MS from 99.4% to 100% and 94.97% to 100%, respectively, while keeping a high specificity (Table 5). Furthermore, a high diagnostic accuracy was revealed when sIgM was replaced by CD200. Similarly, the substitution of FMC7 and CD79b by CD200 in a simplified 4-marker MS has slightly refined the accuracy of MS to 99.8% while keeping high sensitivity and specificity (Table 5).

**Table 5:** Sensitivity, specificity, and accuracy of Matutes scoring systems CLL versus Non-CLL differential diagnosis.

*Scoring system*	*Sensitivity % (95% CI)*	*Specificity % (95% CI)*	*CLL vs*. *non-CLL % (95% CI)*
**CD5, CD23, FMC7, sIgM, CD79b**	94.97 (91.0 - 97.6)	100.0 (92.3 – 100.0)	99.4 (97.4–100.0)
**CD5, CD23, FMC7, sIgM, CD79b, CD200**	100.0 (98.2 – 100.0)	98.04 (89.6 - 100.0)	100.0 (98.4–100.0)
**CD5, CD23, sIgM, CD200**	93.97 (89.7 - 96.8)	100.0 (93.0 - 100.0)	99.8 (98.1–100.0)

## Discussion

Flow cytometry immunophenotyping is crucial for the diagnostic workup of B-CLPD due to its cost-effectiveness and capability of characterizing multiple parameters simultaneously that may enhance the accuracy of flow cytometry-based diagnosis [20]. In the current cohort, we have assessed the value of CD200 in the differential diagnosis of different B-chronic lymphoproliferative disorders and whether it adds a discriminative potential to MS. Although the latter score has been essential for the diagnosis of CLL, in some cases, the diagnosis might be challenging based on the five markers included, which is particularly featured by “atypical CLL” with MS of 3 [21,22]. This would justify the addition of further markers such as CD200, or relying on further evaluation (i.e., FISH) and clinical decision in atypical cases [22–24]. While CD200 evaluation is not required in the CLL diagnostic criteria by the World Health Organization [14], it was implemented in the EuroFlow guidelines and earlier research has declared it’s a high diagnostic value [25].

In concordance with previous results, CLL cases in this cohort showed a consistent manner of CD200 positivity together with the 10 cases of the atypical phenotype (MS <4), though with lower intensity levels [5,23,26]. Additionally, CD200 expression was significantly higher in CLL than in other B-CLPD in agreement with others [10,12,16,17,21]. We have demonstrated that all MCL cases enrolled were negative for CD200 in agreement with most previous studies [13,16,27–29], while others reported a dim expression of CD200 in some MCL [5,6]. Also, we have illustrated that CD200 is expressed in other non-MCL B-CLPD, yet in a heterogeneous pattern, ranging from negative to moderate as featured by others [6,10,29–31].

The contribution of CD200 to modify the accuracy of MS has not been addressed in Iraq with few earlier international studies. A German group (Köhnke et al., in 2017) had incorporated the MFIR of CD200 positive cells into MS and proposed a new score called "CLLflow Score,” with a score above zero correlates with CLL diagnosis, whereas a score of zero or below is unlikely for classical CLL cases [21]. Likewise, two more recent studies (D′Arena et al., 2018) [17], and (Mora et al., 2019) [6] have revealed that CD200≥ 30% has a high sensitivity, specificity and high diagnostic accuracy. Moreover, when CD200 assessed with other MS markers or replaced one or more markers, an increased accuracy of the score was manifested. In agreement with the previous studies, this study has displayed that CD200 (≥30%) incorporation into MS has refined its accuracy to (100%), and therefore, raised its robustness in CLL diagnosis [6,17,21]. It’s worthy to mention that although MFIR is a reliable marker of antigen expression level, and has shown a high diagnostic accuracy (though lower than CD200≥30% in the current study), is not easy to calculate in daily routine cases and not commonly documented in flow cytometry reports, hence not accessible for clinicians [21].

On the other hand, both CD79b and FMC7 have demonstrated a low performance in contrast to what has been described in earlier studies [6,21]. Such variations might be attributed to the study size, and whether a positive marker expression was considered as percentage of positive B-cell population (whether the cutoff point was 20% or 30%) or MFIR. Also if internal control or isotype control was regarded as the negative population in flow cytometry analysis. Finally, factors related to the antibodies comprising the B-CLPD panel, like antibodies conjugated fluorochromes, and their clones might possibly play a role too.

sIgM shown to be a sensitive yet not specific marker in CLL differentiation from other B-CLPD. This might be explained by an aberrantly low expression of sIgM in some B-CLPD with brighter sIgM expression in some CLL cases [21].

The classical MS in the current study has shown to be effective in separating CLL from other B-CLPD once the MS is high (≥4) or low (MS 0–1), while when MS value was between 2 and 3, atypical CLL (MS3,n = 10) overlapped with other B-CLPD scoring 3 (n = 7) (Table 4), [32]. The addition of CD200 to MS has reclassified the atypical CLL into classical CLL and hence allowed clear discrimination from other B-CLPD. Indeed, the MS modifications in this study have derived a high accuracy in CLL diagnosis. Additionally, the simplified four-marker MS has correctly identified 187/199CLL (94%) cases as classical scoring 4/4 with a high specificity and diagnostic accuracy [17], while 12 CLL cases scored 3/4 and hence diagnosed as atypical CLL on the basis of classical MS cutoff, the most reliable scoring system yet [5]. Likewise, 4 cases from other B-CLPD (2MZL, and 2PLL cases) have scored 3/4, though these cases were morphologically distinct from CLL. Besides, all MCL cases scored 1-2/4 in the simplified score with no overlap with atypical CLL cases. Despite the fact that CD200 incorporation to classical MS in the current study has demonstrated 100% accuracy, the 4-marker MS would be much preferred in resource limited settings in an attempt to minimize the markers demanded for classical MS while keeping a high diagnostic performance.

In conclusion, CD200 is an accurate diagnostic marker that is consistently expressed in CLL cases and has potentially distinguished atypical CLL cases from MCL in this study. Furthermore, CD200 assessment within an MS seems to be suitable and convenient for laboratories when the antibodies supply is restricted, and cytogenetic tests are not readily available for challenging cases. Moreover, a cost-effective four -marker panel can be designed with improved accuracy compared to the classical MS. Finally, large prospective studies are prudent to analyze and validate diagnostic accuracy of 4-marker MS with an emphasis on the cutoff point that unequivocally distinguish CLL form other B-CLPD.

## Supporting information

S1 Fig Receiver operating characteristics for CD200 positivity as MFIR. (TIF)

S1 Table Supporting information regarding CD markers used at our study. (DOCX)

S2 Table Features of 10 atypical CLL cases. (DOCX)
