# Supplementary figures and images for: The contribution of CD200 to the diagnostic accuracy of Matutes score in the diagnosis of chronic lymphocytic leukemia in limited resources laboratories
Source: PLoS One. 2021 Feb 19;16(2):e0247491. doi: 10.1371/journal.pone.0247491 (PMC7895405; doi:10.1371/journal.pone.0247491)

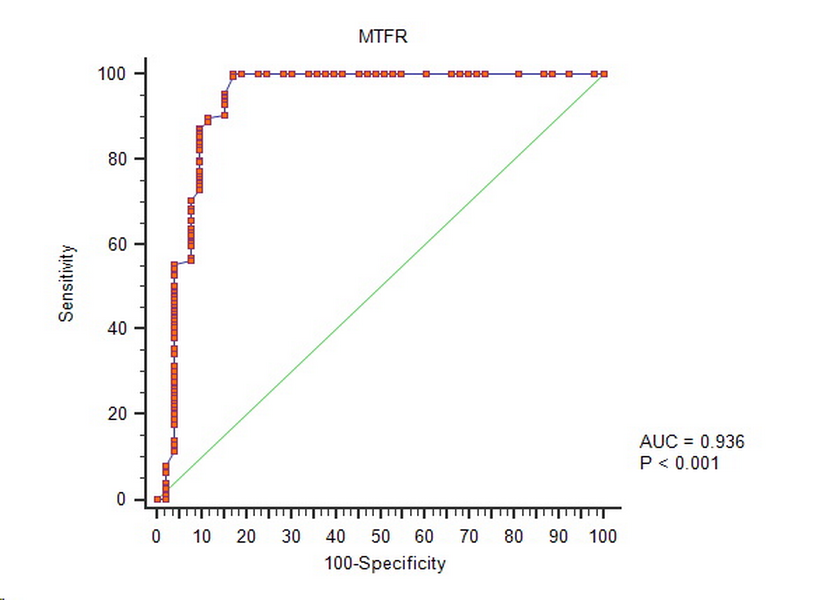

Supplement: S1 Fig — (TIF) [file pone.0247491.s001.tif]
