# Supplementary material for: The contribution of CD200 to the diagnostic accuracy of Matutes score in the diagnosis of chronic lymphocytic leukemia in limited resources laboratories
Source: PLoS One. 2021 Feb 19;16(2):e0247491. doi: 10.1371/journal.pone.0247491 (PMC7895405; doi:10.1371/journal.pone.0247491)
Supplement: S1 Table — (DOCX) [file pone.0247491.s002.docx]

Table S1 Supporting information regarding CD markers used in this study

| **Marker** | **Fluorochromes** | **Clone** | **Dilution** |
| --- | --- | --- | --- |
| CD3 | FITC | SK7 | 1:50 |
| CD5 | FITC | L17F1 | 1:50 |
| FMC7 | FITC | CBVCS-5 | 1:50 |
| CD10 | PE | HI10a | 1:100 |
| CD11c | PE | S-HCL-3 | 1:50 |
| CD19 | APC | H1B19 | 1:100 |
| CD20 | PE | L27 | 1:100 |
| CD23 | PE | CBVCS-5 | 1:100 |
| CD25 | PE | 2A3 | 1:100 |
| CD79b | PE | CB3-1 | 1:50 |
| CD45 | PerCP | 2D1 | 1:50 |
| CD103 | FITC | Ber-ACT8 | 1:50 |
| CD123 | APC | 7G3 | 1:200 |
| CD200 | PE | MRC OX-104 | 1:100 |
| Kappa | FITC | TB28-2 | 1:100 |
| Lambda | PE | 1-155-2 | 1:100 |
| IgM | FITC | G2-127 | 1:100 |

Phosphate buffer saline (BSA) was used for the purpose of antibody titration
