# Supplementary material for: The contribution of CD200 to the diagnostic accuracy of Matutes score in the diagnosis of chronic lymphocytic leukemia in limited resources laboratories
Source: PLoS One. 2021 Feb 19;16(2):e0247491. doi: 10.1371/journal.pone.0247491 (PMC7895405; doi:10.1371/journal.pone.0247491)
Supplement: S2 Table — (DOCX) [file pone.0247491.s003.docx]

Table S2 Features of 10 atypical CLL cases

| **Sample no., age, gender** | **Sample type** | **CD5** | **CD23** | **CD79b** | **FMC7** | **Light chain** | **MS** | **IHC for cyclinD1** |
| --- | --- | --- | --- | --- | --- | --- | --- | --- |
| 1,63,F | PB | + | + | - | + | Moderate | 3 | - |
| 2,45,M | BM | + | - | + subset | + | Dim | 3 | - |
| 3,62,M | PB | + | + | + subset | + | Moderate | 3 | - |
| 4,70,F | BM | + | + | Dim | + | Moderate | 3 | - |
| 5,75,M | PB | + | - | Dim | + | Dim | 3 | - |
| 6,89,F | PB | + | + | - | + | Moderate | 3 | - |
| 7,81,M | PB | + | - | Dim | + | Dim | 3 | - |
| 8,60,M | BM | + | + | Dim | + | Moderate | 3 | - |
| 9,63,M | PB | + | - | + subset | + | Dim | 3 | - |
| 10,69,M | PB | + | + | Dim | + | Moderate | 3 | - |

PB=Peripheral blood, BM =Bone marrow , IHC=Immunohistochemistry ,+=Positive , -= Negative
